# Reliable Dual Tensor Model Estimation in Single and Crossing Fibers Based on Jeffreys Prior

**DOI:** 10.1371/journal.pone.0164336

**Published:** 2016-10-19

**Authors:** Jianfei Yang, Dirk H. J. Poot, Matthan W. A. Caan, Tanja Su, Charles B. L. M. Majoie, Lucas J. van Vliet, Frans M. Vos

**Affiliations:** 1 Quantitative Imaging Group, Department of Imaging Physics, Delft University of Technology, 2628CJ Delft, The Netherlands; 2 Department of Radiology, Academic Medical Center Amsterdam, 1105AZ Amsterdam, The Netherlands; 3 BIGR, Department of Medical Informatics and Radiology, Erasmus MC, Rotterdam, The Netherlands; University of Minnesota, UNITED STATES

## Abstract

**Purpose:**

This paper presents and studies a framework for reliable modeling of diffusion MRI using a data-acquisition adaptive prior.

**Methods:**

Automated relevance determination estimates the mean of the posterior distribution of a rank-2 dual tensor model exploiting Jeffreys prior (JARD). This data-acquisition prior is based on the Fisher information matrix and enables the assessment whether two tensors are mandatory to describe the data. The method is compared to Maximum Likelihood Estimation (MLE) of the dual tensor model and to FSL’s ball-and-stick approach.

**Results:**

Monte Carlo experiments demonstrated that JARD’s volume fractions correlated well with the ground truth for single and crossing fiber configurations. In single fiber configurations JARD automatically reduced the volume fraction of one compartment to (almost) zero. The variance in fractional anisotropy (FA) of the main tensor component was thereby reduced compared to MLE. JARD and MLE gave a comparable outcome in data simulating crossing fibers. On brain data, JARD yielded a smaller spread in FA along the corpus callosum compared to MLE. Tract-based spatial statistics demonstrated a higher sensitivity in detecting age-related white matter atrophy using JARD compared to both MLE and the ball-and-stick approach.

**Conclusions:**

The proposed framework offers accurate and precise estimation of diffusion properties in single and dual fiber regions.

## Introduction

Diffusion-weighted magnetic resonance imaging (DW-MRI) can provide unique information about the integrity of white matter structures in the brain. Conventionally, the diffusion is described by a single rank-2 diffusion tensor [1], which is estimated from diffusion-weighted images (DWIs). There is an ongoing debate on how to effectively characterize the diffusivities in voxels containing complex anatomical structures such as crossing fibers. In these voxels the diffusion profile is not adequately described by a single rank-2 tensor [2,3].

Several sophisticated models for the diffusion in white matter have shown the potential to estimate more plausible anatomical properties of complex tissue structures, for instance the ‘ball-and-stick’ model [4], the composite hindered and restricted model of diffusion (CHARMED)[5], and the neurite orientation dispersion and density imaging approach (NODDI) [6]. Although many more techniques were proposed, the aforementioned techniques relate to our approach. A comprehensive overview is presented in [7].

The ‘ball-and-stick’ approach models the diffusion by one isotropic compartment and a set of linear, 1D diffusion profiles. The method is widely applied and works well for reconstructing the orientations of fiber bundles [8], even though these fiber bundles are not represented by full, 3D diffusion profiles.

CHARMED explicitly models the apparent slow diffusing component arising from *restricted*, intra-axonal diffusion (a non-Gaussian process). It yielded unbiased estimation of the orientations of two or more fiber compartments with low angular uncertainty. However, the application of CHARMED is challenging due to measurements at very high b-values causing both signal-to-noise ratio and scanning time limitations.

A clinically feasible technique for in vivo neurite orientation dispersion and density imaging (NODDI) [6] was proposed. NODDI adopts a tissue model that distinguishes three types of microstructural environment: intra-cellular, extra-cellular, and cerebrospinal fluid (CSF) compartments. The signal of intra-cellular diffusion is described by zero radius cylinders [9] (like in the ball-and-stick approach); the extra-cellular part is modeled by anisotropic, Gaussian diffusion and the CSF compartment is modeled as isotropic Gaussian diffusion. Experiments showed that quantities derived from NODDI such as the neurite orientation dispersion provided more specific markers of brain tissue microstructure than standard indices from classical, single-tensor DTI [6].

Other methods [10] [11] [12] were proposed that also aim to recover specific tissue parameters from the diffusion signal, such as cell size and cell density. However, these models are not directly compartment-specific. Instead, a multiple-tensor model [13] describes the complex, full diffusion shape by a weighted sum of 3D tensors. We mean with full diffusion shape the 3D probability density function representing the position of water molecules after a certain diffusion time. We assume that an anisotropic Gaussian distribution (mathematically a rank-2 tensor model) accurately approximates this distribution in single fiber voxels. The ball-and-stick model represents the diffusion as a line-like profile, thereby ignoring a ‘lateral’ diffusion component. This is an important difference because pathology leads to an increase of the lateral diffusivity, which can be measured with our representation. The model is an intuitive, physical representation and a natural extension of the classical single tensor. Also, it does not necessarily require extremely high b-values. Previously, we introduced an optimization framework that rendered a constrained dual tensor model (DTM) as well as a set of diffusion weighting parameters, such that both the diffusion *shape* and the diffusion *orientations* of crossing fibers could be accurately estimated[3].

Unfortunately, even a constrained DTM is prone to overfitting in areas containing a single fiber bundle, causing biased volume fractions and imprecise diffusivity estimates. As such, a new challenge arises: how to automatically adapt the model complexity to warrant an accurate characterization of the underlying diffusion processes?

Many model selection methods were introduced in the DWI field, for instance based on constrained spherical deconvolution (CSD) [14], the Bayesian information criterion (BIC) and the generalization-error [15] A limitation of the CSD approach is that it requires tuning of a threshold to reject small contributions. Furthermore, BIC is influenced by the estimated likelihood and non-estimated factors such as the number of parameters and the sample size. The generalization-error method is a non-local model selection technique. Importantly, all these approaches involve model selection techniques that make hard decisions to select an appropriate model.

Automatic relevance determination (ARD) aims to eliminate the redundant parameters in a complex model, such that the simplified model yields a better description of the data [16]. Behrens [8] adopted ARD for assessing the appropriate number of fiber orientations in each voxel for fiber tracking. This method ensures that if there is no evidence for a second fiber orientation in the data, the volume fraction attributed to this fiber will automatically be forced to zero. ARD methods assume a prior distribution for the model parameters. A Gaussian distribution is a straightforward choice for a prior[16]. Such a prior may involve hyper-parameters to tune its shape. Previous ARD approaches [8,17,18] involved marginalization (integration) over the hyper-parameters to get a prior for each parameter separately. Such a prior is likely to be suboptimal for different diffusion geometries since potential correlations between the parameters of complete models are ignored.

We present a new framework for data-acquisition adaptive estimation of the diffusion shape of simple *and* complex white matter structures. We consider the method data-acquisition adaptive as it takes properties of the data-acquisition into account such as the number of gradient directions, the number of b-values used and the noise level. The method is based on ARD for a rank-2 dual tensor model and assesses whether two anisotropic tensors are ‘mandatory’ to model the acquired diffusion-weighted signals. Our ARD estimates the mean of the a posteriori distribution, i.e. the model parameters given the data, exploiting Jeffreys prior [19] [20]: ‘JARD’. This data-acquisition adaptive prior is based on the Fisher’s information matrix [21]. Previous work on ARD for diffusion-weighted MRI primarily focused on the accurate reconstruction of fiber *orientations* based on the ball-and-stick model [8,17]. This rank-1 tensor model is not appropriate for estimating the diffusion shape as reflected by a rank-2 tensor model. The proposed JARD method is particularly suited for application in comparative studies in which the goal is to assess subtle differences in diffusion *shape* between patients and matched controls.

## Methods

The JARD framework for estimation of the diffusion shape processes every voxel in the same way. It estimates the parameters of a constrained dual tensor model (DTM) by computing the mean of the posterior distribution sampled by a Markov Chain Monte Carlo (MCMC) approach. The algorithm is initialized by applying the constrained DTM to the measured diffusion-weighted signals using maximum likelihood estimation (MLE). The prior on the parameters in the MCMC sampling is based on the non-informative Jeffreys prior. This prior forces parameters, particularly the volume fraction, towards zero when there is little to no information in them. This will be verified experimentally in the Experiments and Results section.

### Dual Tensor Diffusion Model

We assume that the diffusion in every fiber bundle is mono-exponential and Gaussian. The diffusion-weighted signal in all voxels is initially modeled by a so-called dual tensor model (DTM) [3]. This model contains signal contributions of up to two fiber bundles and an isotropic component and is given by

$$S_{\theta ,j}=S_{0}\left(\sum_{i\in \left\{1,2,iso\right\}}f_{i}\exp(-b_{j}g_{j}^{T}D_{i}g_{j})\right),$$ (1)

where *S*_**θ**,*j*_ is the diffusion-weighted signal given parameter vector **θ** for diffusion weighting *b*_*j*_ in gradient direction **g**_*j*_ and *S*_0_ the signal without diffusion weighting. **D**_1_ and **D**_2_ are rank-2 tensors to model the anisotropic diffusion in each fiber, **D**_*iso*_ is the amount of isotropic diffusion (i.e., *D*_*iso*_⋅**I**_3×3_, *D*_*iso*_ representing the scalar amount of isotropic diffusion), and *f*_*i*_ represents the volume fraction of component **D**_*i*_. Note that the DTM in Eq (1) reduces to a single tensor model (STM)–reflecting a single fiber–if *f*_1_ > 0 and *f*_2_ = 0 or vice versa. The volume fraction parameters play an essential role in our JARD scheme.

### Maximum Likelihood Estimation of a constrained DTM

The measured diffusion weighted image (DWI) ${\tilde{\text{S}}}_{j,\sigma }$ with diffusion weighting *b*_*j*_ in direction **g**_*j*_ is corrupted by Rician noise of standard deviation *σ* [22]. Therefore, the probability density function (PDF) for ${\tilde{\text{S}}}_{j,\sigma }$ is given by

$$p({\tilde{\text{S}}}_{j,\sigma }|\theta )=\frac{{\tilde{\text{S}}}_{j,\sigma }}{\sigma^{2}}\exp\left(-\frac{{\tilde{\text{S}}}_{j,\sigma }{}^{2}+S_{\theta ,j}{}^{2}}{2\sigma^{2}}\right)I_{0}\left(\frac{{\tilde{\text{S}}}_{j,\sigma }S_{\theta ,j}}{\sigma^{2}}\right),$$ (2)

with *I*_0_(⋅) the zero-th order modified Bessel function of the first kind. The DWIs are statistically independent, so that the joint probability density function $p({\tilde{S}}_{\sigma }|\theta )$ of the signal profile ${\tilde{S}}_{\sigma }$ is given by the product of the marginal distributions for the measured signals ${\tilde{\text{S}}}_{j,\sigma }$ in each of the *N*_*g*_ diffusion weighted directions **g**_*j*_:

$$p\left({\tilde{S}}_{\sigma }|\theta \right)=\prod_{j=1}^{N_{g}}p\left({\tilde{\text{S}}}_{j,\sigma }|\theta \right)$$ (3)

Here, $p({\tilde{S}}_{\sigma }|\theta )$ is the likelihood function of **θ** given ${\tilde{S}}_{\sigma }$. The underlying parameter values can be inferred by maximizing this likelihood function [23]

$${\hat{\theta }}_{\text{MLE}}=\arg\;\max_{\theta }\left\{p({\tilde{S}}_{\sigma }|\theta )\right\}.$$ (4)

Maximum likelihood estimation (MLE) has a number of favorable statistical properties in the estimation of diffusion properties in crossing bundles [3]. First, under very general conditions, MLE asymptotically reaches the Cramér-Rao lower bound (CRLB). The CRLB is a theoretical lower bound on the variance of any unbiased estimator. Second, the MLE is consistent, which means that it asymptotically (*N*_*g*_ → ∞) converges to the true value of the parameter in a statistically well-defined way [24].

The dual tensor model given in (1) should be parameterized such that its parameter values reside in a well-defined range. In previous work [3], we parameterized the tensor **D**_*i*_ as follows: $D_{i}=R_{i}^{T}E_{i}R_{i}$, where **E**_*i*_ = diag(*λ*_*i*,//_,*λ*_*i*,⊥1_,*λ*_*i*,⊥2_) is a diagonal matrix with the eigenvalues of the tensor **D**_*i*_ on its diagonal. The non-negativity constraint that is imposed on the estimated diffusivity values is accomplished by employing an exponential mapping [3]. The matrices **R**_*i* = 1,2_ describe rotations around the *x*–, *y*– and *z*–axes: **R**_*i*_(*α*_1–4_) = **R**_*x*_(*α*_1_)**R**_*y*_(*α*_2_)**R**_*z*_(*α*_3_±*α*_4_/2). The first two rotations **R**_*x*_(*α*_1_)**R**_*y*_(*α*_2_) determine the orientation of the plane in which the first principal eigenvectors of both tensor reside. **R**_*z*_(*α*_3_ + *α*_4_/2) and **R**_*z*_(*α*_3_ − *α*_4_/2) denote the in-plane rotations of the first principal eigenvector of the two tensors. As such, the parameter vector to be estimated for a dual-tensor model MLE becomes

$$\theta =\{\underset{\text{volume fractions}}{\underset{︸}{f_{1},f_{2},f_{iso}}},\underset{\mathrm{diag}(E_{1})}{\underset{︸}{\lambda_{1,//},\lambda_{1,⊥1},\lambda_{1,⊥2}}},\underset{\mathrm{diag}(E_{2})}{\underset{︸}{\lambda_{2,//},\lambda_{2,⊥1},\lambda_{2,⊥2}}},D_{iso},\underset{\text{fiber orientations}}{\underset{︸}{\alpha_{1},\alpha_{2},\alpha_{3},\alpha_{4}}}\}.$$ (5)

However, MLE does not necessarily yield useful estimates. A potential error in the estimated parameters is greatly influenced by the degrees of freedom (DOFs) and the covariance(s) between parameters. We demonstrated that restricting the DOFs by imposing constraints on the DTM greatly reduces the covariance between parameters. The experiments in [3] showed that precise and accurate estimation can be achieved if we apply the following constraints:

$$\lambda_{1,//}=\lambda_{2,//},$$ (6)

$$\begin{array}{l} \lambda_{1,⊥1}=\lambda_{1,⊥2}\equiv \lambda_{1,⊥}\\ \lambda_{2,⊥1}=\lambda_{2,⊥2}\equiv \lambda_{2,⊥} \end{array},$$ (7)

$$D_{iso}=C_{free-water},$$ (8)

$$f_{1}+f_{2}+f_{iso}=1.$$ (9)

Eq (6) imposes that the “unrestricted” diffusivity (i.e., free diffusivity) along the fibers, denoted by the first eigenvalues of **D**_1_ and **D**_2_, are equal. Eq (7) states that the diffusion perpendicular to the fiber orientation is assumed to be axially symmetric, which models the average shape of axons. Eq (8) defines that *D*_*iso*_ equals *C*_*free–water*_ = 3×10^−3^ *mm*^2^*s*^−1^, the diffusivity of free water at body temperature 37°C, and Eq (9) states that the two anisotropic tensors plus the isotropic compartment fill the entire volume of each voxel.

Constraining the DTM cannot avoid the inherent risk of overfitting. This happens when a complex model is fitted to simple data, e.g. fitting multiple tensors to data of a single fiber bundle. Typically, this yields an increase of the variance and the covariance of the parameters, but also leads to biased diffusivity estimates.

### Automatic Relevance Determination

Bayes factors offer an alternative to model selection by the classical likelihood test [25]. It computes the evidence for a model to be used in model selection. However, calculating the evidence for any model requires integration over all model parameters, weighted by the parameter priors. This is computationally unfeasible, especially with high-dimensional parameter spaces for which no analytical solution exists. ARD was introduced exactly to cope with such issues [16] [8] [17] [26] [27]. Compared to the Bayes factors approach, ARD does not fit competing potential models to the data and compares them on the basis of the residual after fitting. Instead, ARD always fits a complex model to the data and forces irrelevant parameters to zero, so that a complex model reduces to a simpler one.

Our JARD estimates the mean of the posterior distribution of the constrained DTM based on Bayes’ theorem [16]. The posterior distribution $p(\theta |{\tilde{S}}_{\sigma })$ is

$$p(\theta |{\tilde{S}}_{\sigma })=\frac{p({\tilde{S}}_{\sigma }|\theta )p(\theta )}{p({\tilde{S}}_{\sigma })},$$ (10)

where $p({\tilde{S}}_{\sigma }|\theta )$ is the aforementioned likelihood function, *p*(**θ**) the prior probability of **θ** in the DTM, and $p({\tilde{S}}_{\sigma })$ the evidence for the DTM. As the evidence term in Eq (10) is constant for any measured signal, the posterior probability distribution in JARD becomes

$$p(\theta |{\tilde{S}}_{\sigma })\propto p({\tilde{S}}_{\sigma }|\theta )p(\theta )$$ (11)

Our framework estimates the posterior distribution given the data, which is influenced by the likelihood function and the prior. We introduce a data-acquisition adaptive prior for DTM parameters based on Jeffreys theorem (see next subsection). It allows simplifying a complex model to a simple model by automatically forcing volume fractions, which are not supported by data to zero.

JARD employs a Markov Chain Monte Carlo (MCMC) technique with Metropolis-Hasting sampling of the posterior distribution $p(\theta |{\tilde{S}}_{\sigma })$ [28] [29]. The MCMC draws 5000 samples from the posterior distribution in the nine-dimensional parameter space. The algorithm is listed in Algorithm 1. It is initialized by MLE of the constrained DTM. The final JARD estimate ${\hat{\theta }}_{JARD}$ is the mean of 3000 accepted samples after a burn-in period of 2000.

If the posterior estimates of *both* anisotropic fractions lie in a small interval around their MLE value, then this would indicate that the prior did not significantly change the outcome and that fitting the initial dual tensor model was justified. Reversely, if the posterior estimate for *one* of the two anisotropic fractions does not significantly differ from zero, then its corresponding tensor compartment can be treated an unnecessary parameter. In such a case, the estimation essentially returns a ‘single-tensor’ model.

### Jeffreys Prior

Methods to choose the prior for a Bayesian analysis can be divided into two groups: informative and non-informative priors [27] [30]. An informative prior expresses specific, definite information about a variable, whereas an uninformative (or diffuse) prior expresses only general information about a variable. We aim to introduce a new, data-acquisition adaptive prior, which makes JARD non-informative. Specifically, we adopt Jeffreys non-informative prior *p*_*J*_(**θ**) which can be written as:

$$p_{J}(\theta )\propto \det{\left(I(\theta )\right)}^{1/2},$$ (12)

where **I**(**θ**) denotes the Fisher information matrix given by

$$I(\theta )=-E_{S}\left\{\frac{\partial^{2}\;\ln(p(S|\theta ,\sigma ))}{\partial \theta \partial \theta^{T}}\right\}.$$ (13)

The Fisher information matrix **I**(**θ**) provides the amount of expected information about the parameter vector **θ** in measurements. By definition, it is influenced by properties of the data-acquisition such as the number of data points and the noise. In general, Jeffreys prior is in agreement with one’s intuition that if a parameter is necessary, it must be supported by the data. Poot [31] showed that the Fisher information matrix for Rice distributed measurements given by Eq (13) can be efficiently computed.

We aim to exploit Jeffreys prior in Bayesian estimation to discriminate between configurations that yield a degenerate or a non-degenerate model. The preferred prior must convey support for a dual tensor representation in crossing fibers, e.g. represented by two tensors at 90-degree angles. Therefore, the prior should be flat in such a configuration, making that it only mildly affects she posterior distribution, which would peak near the initial dual tensor parameters obtained by MLE. Reversely, consider an actual single tensor configuration, e.g. a dual tensor model consisting of two tensors with the same shape and orientation and having *f*_1,2_ = 0.45. In this case the dual-tensor model is degenerate and the prior must be harsh, promoting a near-zero volume fraction in the posterior distribution.

In order to meet these preferred properties, we use a prior based on Jeffries prior:

$$p(\theta )=\det{\left(I(\theta )\right)}^{-1/2},$$ (14)

**Fig 1:**
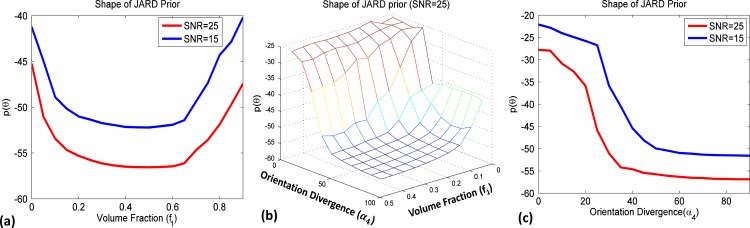
Plots of the prior probability for a model of two crossing fibers (detailed parameters, see Table 1). a) Prior probability for crossing fibers at 90-degree angle as a function of *f*_1_. b) Prior probability as a function of both *f*_1_ and *α*_4_. c) Prior probability for crossing fibers of equal volume fraction as a function of *α*_4_. The blue and red curves in subplots (a) and (c) were generated using respectively SNR = 15 and SNR = 25.

Fig 1 illustrates the shape of the prior. Notice in Fig 1(A) that as the volume fraction *f*_1_ ≈ 0.45 the prior is rather flat. This is because in a true dual tensor configuration, there is no correlation between the model parameters, as such yielding a maximum for det(**I**(**θ**)). Reversely, as the volume fraction *f*_1_ approaches zero to establish a single tensor configuration, there will be large correlation between several parameters yielding a very small det(**I**(**θ**)) and in turn a sharp rise in the prior probability. Additionally, Fig 1(B) shows that as orientation divergence *α*_4_ decreases to zero, the prior increases in the direction of *f*_1_ = 0. As such, the prior will favor a ‘simpler’ configuration (i.e. push *f*_2_ to zero) as long as the decrease in goodness of fit is more than compensated for by an increase in the prior probability. Note that the bathtub shape of the prior probability in Fig 1(A) indicates that the roles of the two volume fractions can be exchanged. The deviation from a pure symmetric curve is due to the difference in FA of the two crossing fiber bundles.

A nice property of Jeffreys prior is that it automatically adapts to data acquisition conditions. This is exactly why we describe our prior as data-acquisition adaptive. For example, a decreased number of gradient directions or an increased noise level will reduce the Fisher information of a dual tensor model. This corresponds to larger theoretical minimum variance and a reduced support of that model. Therefore, the curve representing the lower SNR in Fig 1(C) (blue) starts to increase at a larger *α*_4_ than that corresponding to the higher SNR (red). This shows that a higher SNR yields a better orientation sensitivity in detecting and estimating crossing fibers. In effect, our prior enforces that the dual-tensor model reduces to the single tensor representation unless there is sufficient support for two non-zero tensors in the data. Furthermore, a higher b-value by itself enhances the support for a two-tensor representation (see [13] [32] [33]). However, such a higher b-value usually comes with a lower signal to noise ratio. Therefore, the effect of varying the b-values is more difficult to predict. In previous work, we found that imaging at two b-values (1000, 3000) is appropriate for precise estimation of diffusion parameters in fiber crossings.

**Algorithm 1. Markov Chain Monte Carlo with Metropolis-Hastings sampling**

Algorithm for estimating the mean of a multivariate posterior distribution with our prior using a Markov Chain Monte Carlo method employing Metropolis-Hastings sampling. The ‘proposal’ distribution *Q*(**θ**'|**θ**_*t*_) = **θ**_*t*_ + **Δ**⋅**N**(0,1) with **N**(0,1) denoting a multivariate Gaussian distribution with zero mean and standard variance 1; and **Δ** the step size for parameter vector **θ**. The step sizes for all parameters are: 10^−5^ (with unit *mm*^*2*^*s*^*-1*^) for the diffusivity parameters, 10^−2^ (with unit *rad) for* the angles, and 10^−2^ for the volume fractions.

**For** all voxels

        $\theta_{0}={\hat{\theta }}_{MLE}$ *// Initialize vector*

        **For** t = 0 to N-1

            **θ**' = *Q*(**θ**'|**θ**_t_) // Draw candidate from ‘proposal’ distribution

            *p*(**θ**') = det(**I**(**θ**'))^−1/2^ // Calculate our prior

            $p(\theta '|\tilde{S})=p(\tilde{S}|\theta ')p(\theta ')$ // Calculate posterior probability

                α = p($\theta '|\tilde{S}$)/p($\theta_{\text{t}}|\tilde{S}$) // Calculate the acceptance ratio

                **If** α > = 1 then

                        **θ**_t+1_ = **θ**' // Accept the candidate vector

                **Else**

                        r = *U*(0,1) // Draw random variable r between 0 and 1;

                        **If** r < = α

                                **θ**_*t*+1_ = **θ**' // Accept the candidate vector

                        **Else**

                                **θ**_*t*+1_ = **θ**_*t*_; // Keep the previous vector

                        **Endif**

                **Endif**

        **Endfor**

        ${\hat{\theta }}_{ARD}=\frac{1}{N-N_{burn-in}}\sum_{t=N_{burn-in}}^{N-1}\theta_{t}$ // Compute mean of accepted samples after burn-in

**Endfor**

## Results

All experiments below were carried out on a DELL laptop computer with an Intel i7-2620CPU @2.7GHz and 4 GB RAM running a Windows-7 64-bit operating system. The method was implemented in MATLAB_R2014b. The average execution time on our brain image data was 6 voxel/s. Applying our method to one DTI volume (from dataset A, see below) took approximately one day by parallel processing of voxels on a cluster of 12 Intel Pentium cores. Clearly, a further speedup can be achieved by involving more cores because the execution time decreases linearly with the number of cores. Furthermore, the presented number is measured based on interpreted MATLAB code. We expect that a further speedup can be achieved by compiling the MATLAB code.

In the first part of this section we evaluate the performance of estimating the parameters of our constrained dual tensor model by JARD and by MLE on simulated data. We studied the differences between JARD and MLE as a function of the volume fraction for simulated crossing fibers under realistic conditions. Diffusion measurements were simulated by means of the model presented in Eq (1). The parameters of crossing fibers are listed in Table 1 and are in agreement with the work of Pierpaoli [34] who reported diffusivities ranging from 0.25×10^−3^ to 1.5×10^−3^ *mm*^−2^ *s*. At the given diffusivities *FA*_1_ = 0.91 and *FA*_2_ = 0.67. The SNR (defined by *S*_0_/*σ*) was 25 [3]. Two measurements at *b* = 0 *mm*^-2^ *s* were simulated. Furthermore, 92 gradient directions were adopted for each of two *b*-values (1.0⋅10^3^*mm*^−2^*s* and 3.0⋅10^3^*mm*^−2^*s*), homogeneously distributed over the surface of a sphere. These settings are identical to dataset B (see below) and equal to those reported in [3].

**Table 1:** Model parameters for generating synthetic data. The units of the diffusion parameters *λ* are 10^−3^ *mm*^*2*^*s*^*-1*^.

Parameters	Value (*θ*)
*λ*_1//_	1.480
*λ*_1,⊥1_	0.15
*λ*_1,⊥2_	0.12
*λ*_2//_	1.400
*λ*_2⊥1_	0.4
*λ*_2,⊥2_	0.38
*α*_1,2_	Random
*α*_3_	0.8 *π*
*α*_4_	Variable
*f*_1_	Variable
*f*_*iso*_	0.1
*D*_*iso*_	3.0
*S*_0_	250
*σ*	10

In the second part of this section we demonstrate the potential of the proposed framework for some neuroimaging applications. Therefore, we evaluated the performance on varying types of brain datasets to verify whether a reliable estimation could be achieved. We applied JARD and MLE to the genu of the corpus callosum (CC) representing a single fiber region enclosed at both ends by a crossing with the corticospinal tract (CST). Subsequently, we show a neuroimaging application of our proposed framework, i.e. automatic estimation of diffusion properties.

Three different datasets, acquired with different acquisition protocols, were adopted to explore the two methods. Dataset A concerned diffusion data from one subject of the Human Connectome Project (HCP) [35]. The relevant acquisition parameters of this dataset were: three *b*-values 1000, 2000 and 3000 *s/mm*^*2*^, 90 gradient directions per *b*-shell and three measurements at *b* = 0 per *b*-shell, TE/TR 89.5/5520 *ms*, voxel size 1.25×1.25×1.25 *mm*^*3*^. Dataset B was acquired from one control subject (see also [3]). The acquisition parameters: two *b*-values 1000 and 3000 *s/mm*^*2*^, 92 gradient directions per *b*-shell and one measurements at *b* = 0 per *b*-shell, TE/TR 84/3800 *ms*, voxel size 1.7×1.7×2.2 *mm*^*3*^. Dataset C consisted of data from 24 healthy controls from an ongoing DTI study into the effects of HIV on the brain [36]. The acquisition parameters were: two *b*-values at 1000 and 2000 *s/mm*^*2*^, 64 gradient directions *b*-shell and one measurements at *b* = 0 per *b*-shell, voxel size 2.0×2.0×2.0 *mm*^*3*^. The SNR for each of the three datasets was found to be higher or equal than 20. This was determined by fitting a single tensor model to a selected region of the CC, after which we took the ratio between *S*_**υ**,0_ and the model residual as the estimated SNR.

### JARD versus MLE: simulation experiment

The volume fraction of the constituting compartments of a dual tensor model is a crucial parameter for the modeling of simple and complex fiber geometries. Therefore, we evaluated the performance of the methods as the volume fractions of the two compartments were varied. To assess the robustness for variations in volume fraction, for each volume fraction and divergences of 90 degrees and 45 degrees we generated 100 realizations with the parameters given in Table 1. For each realization ${\hat{\theta }}_{JARD}$ and ${\hat{\theta }}_{MLE}$ were computed.

**Fig 2:**
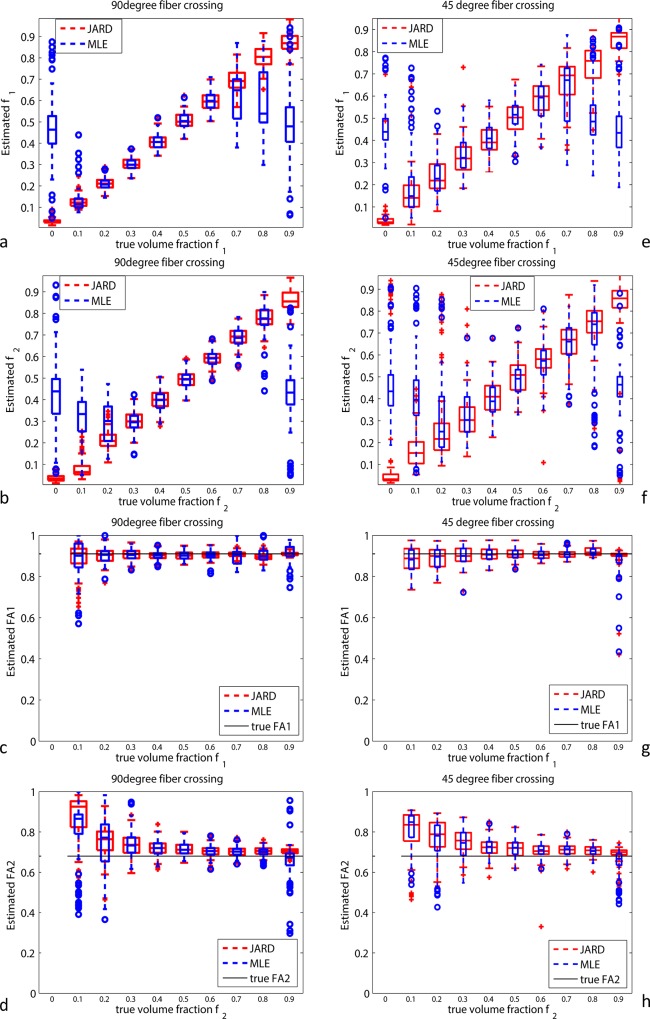
Results of dual tensor model estimation by JARD (red) and MLE (blue) on 100 noisy realizations (SNR 25) of two crossing fibers (divergence: 90 degrees in a-d and 45 degrees in e-h) as a function of volume fraction. The boxplots show: a) and e) volume fraction of the first compartment; b) and f) volume fraction of the second compartment; c) and g) fractional anisotropy (FA) of the first component; d) and h) FA of the second compartment. The simulated model listed in Table 1 and acquisition parameters accords with Dataset B. The boxes display the median and 25th, respectively 75th percentiles of the data distribution; whiskers extend to 1.5 times the interquartile range; values outside these ranges are indicated as individual points.

Fig 2 shows boxplots depicting the results of dual tensor estimation by JARD (red) and MLE (blue). Since the estimation procedure assigns a random label to the first and second tensor, we need to assign the two estimated tensors to the corresponding ground truth compartment. The estimated tensors are sorted by tensor similarity based on the Frobenius norm: the tensor with the smallest Frobenius norm with respect to the ground truth of compartment 1 received the label 1. Fig 2A–2D) show the results for the 90° crossing. The estimated volume fraction by JARD nearly ideally correlates with the ground truth over the entire range of volume fractions, both for single fiber (*f*_1_ = 0 ∨ *f*_1_ = 0.9) and crossing fiber configurations (*f*_1_ ∈ (0, 0.9)), see Fig 2A and 2B). Clearly, MLE yields a random *f*_1_ as the true volume fraction approaches that of a single fiber configuration.

Fig 2(C) shows that the median *FA*_1_ estimation is almost identical for both estimation methods in crossing fiber configurations, irrespective of the volume fraction. Fig 2(D) shows that the estimated *FA*_2_ converges (almost) to the true value as the true *f*_2_ increases. The *FA* estimation in single fibers (*f*_1_ = 0.9 ∨ *f*_2_ = 0.9) appears equally unbiased for both methods, but a *considerably larger spread* is encountered with MLE than with JARD. Note that the estimated *FA*’s with *f*_1_ = 0 ∨ *f*_2_ = 0 essentially represent degenerate measurements and are therefore not shown in the graphs. In the absence of a ground truth this can be detected with JARD as the corresponding volume fraction is automatically forced to (near) zero (see Fig 2 (A)). MLE does not offer such a mechanism, which might lead to a fictitious fiber compartment.

Fig 2E–2H) show the results for the 45° crossing. This figure shows similar trends as obtained for the 90° crossing in Fig 2A–2D), albeit with a larger spread. Specifically, for the 45° crossing JARD yielded *f*_1_ = 0.05 ± 0.06, *f*_2_ = 0.87 ± 0.06 (mean ± standard deviation) at a true *f*_1_ of 0.0. Instead, MLE gave *f*_1_ = 0.47 ± 0.14, *f*_2_ = 0.44 ± 0.15 at true *f*_1_ = 0.0. JARD yielded *FA*_1_ = 0.89 ± 0.07 at a true *f*_1_ = 0.1 while *FA*_2_ = 0.68 ± 0.06 as the true *f*_2_ = 0.9. Alternatively, MLE gave *FA*_1_ = 0.89 ± 0.09 at true *f*_1_ = 0.1 while *FA*_2_ = 0.64 ± 0.08 as the true *f*_2_ = 0.9. Notice that there is not a significant bias in the FA of a tensor if its volume fraction is larger than 0.2. In particular, the *FA* of a tensor is not biased in case the other component has zero volume fraction, i.e. the single fiber situation. Only in the case where there is a small second component, particularly with true *f*_2_ = 0.1 … 0.2, the FA of this tensor is slightly biased.

**Fig 3:**
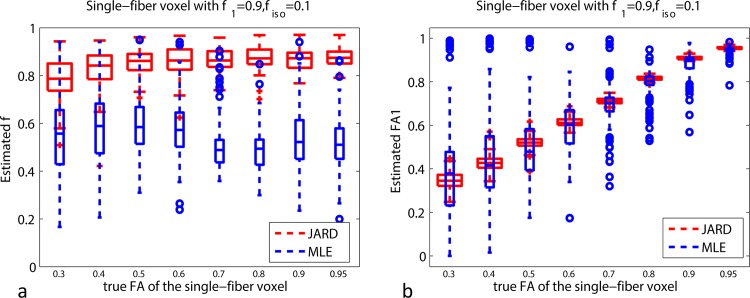
Results of dual tensor model estimation by JARD (red) and MLE (blue) on 100 noisy realizations (SNR 25) of a single fiber and an isotropic compartment as a function of FA. a) Volume fraction of the first compartment; b) Fractional Anisotropy (FA) of the first component. The simulated model are listed in Table 1 and acquisition parameters accords with Dataset B.

The performance of the two methods in single fiber regions is further corroborated in Fig 3. It shows the dual tensor model estimation by JARD (red) and MLE (blue) on a single fiber with a small isotropic compartment while only the single fiber’s *FA* is varied.

Fig 3(A) shows that the estimated *f*_1_ with JARD improves with increasing FA and approximates the ground truth. MLE essentially yields a random estimate of *f*_1_ irrespective of the actual FA. Fig 3(B) confirms that the estimation of *FA*_1_ remains largely unbiased with both methods. Clearly, the spread in the estimated *FA*_1_ with MLE is much larger than with JARD. Particularly, JARD yielded f_1_ = 0.79 ± 0.08 at a true FA = 0.3 and f_1_ = 0.87 ± 0.04 at a true FA = 0.95 (true f_1_ = 0.9). Instead, MLE gave f_1_ = 0.56 ± 0.17 at true FA = 0.3 and f_1_ = 0.52 ± 0.11 at true FA = 0.95. Furthermore, JARD yielded FA_1_ = 0.35 ± 0.04, at a true FA = 0.3 and FA_1_ = 0.95 ± 0.01, at a true FA = 0.95. Similarly, MLE gave FA_1_ = 0.38 ± 0.23, at a true FA = 0.3 and FA_1_ = 0.95 ± 0.02, at a true FA = 0.95.

The effect of varying the fiber orientation was assessed by generating diffusion measurements through Eq (1) using the parameter values given in Table 1, with *α*_4_ ∈ {0, 20°, 40°, 90°}. For each such configuration 100 noisy realizations were generated. Subsequently, the model parameters were estimated by means of MLE and ARD using both the prior from Behrens’ paper (i.e. *f*^−1^(1−*f*)^−1^) as well as Jeffreys prior, referred to as BARD and JARD respectively.

**Fig 4:**
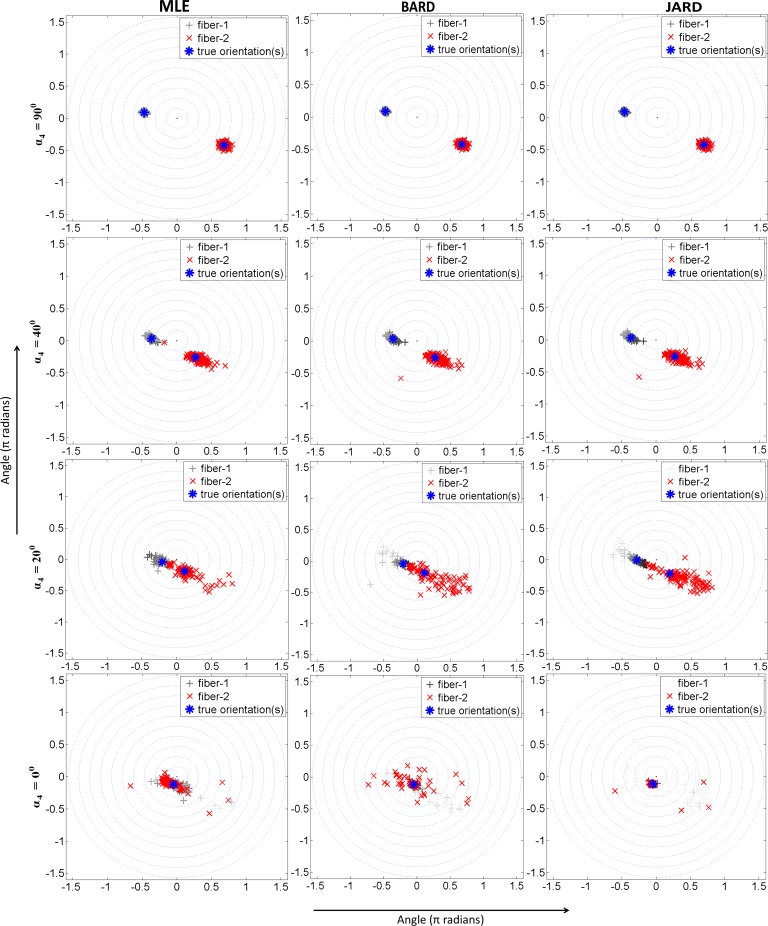
Scatterplots of estimated orientations by MLE and ARD using Behrens prior (BARD) and Jeffreys prior (JARD) for with varying *α*_4_. The two axes in each subplot represent angles (unit: π rad). The orientation of the tensor with the largest volume fraction is shown in red, the other in gray. The intensity of each symbol is scaled by the volume fraction of the tensor.

The outcome of this experiment is presented in Fig 4. It contains scatterplots of estimated orientations of the largest eigenvectors of the tensors.

The results confirm that the three methods yield very similar performance as *α*_4_ ≥ 20°. However, the proposed JARD clearly yields the most precise orientation estimation if *α*_4_ = 0: the tensors with a large volume fraction assert a single orientation, while the scattered orientations all have a very small volume fraction.

Summarizing, the graphs demonstrate two things: 1) JARD facilitates accurate estimation of volume fractions especially with increasingly unbalanced real volume contributions, in which case MLE grossly fails; 2) the estimation of FA by JARD shows a much narrower distribution than by MLE, especially in single fibers

### JARD versus MLE: brain imaging

**Fig 5:**
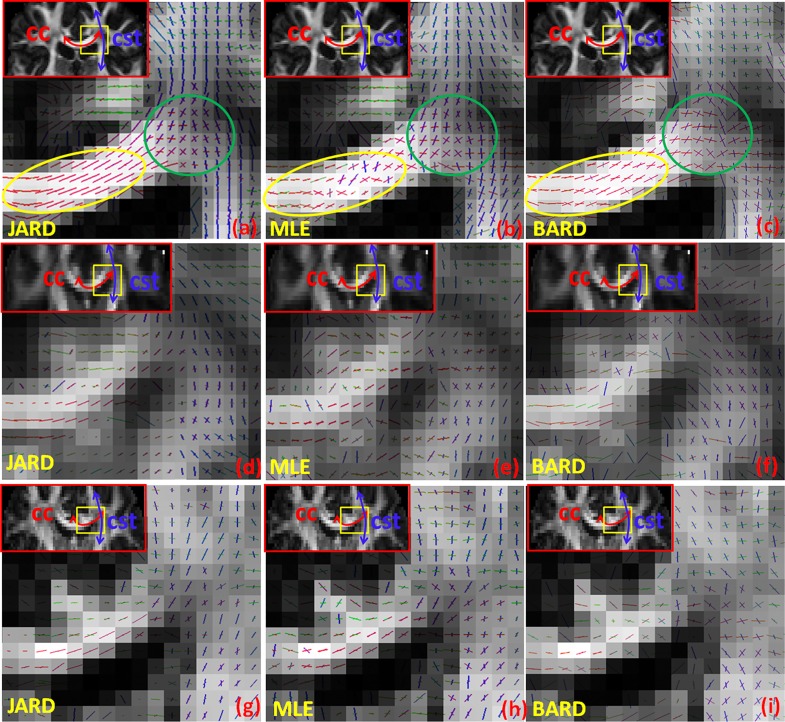
Results of our JARD (a, d, g), MLE (b, e, h) and FSL-ARD using the ball-and-stick model (c, f, i) in a region where the corpus callosum (CC) crosses the corticospinal tract (CST) in three randomly selected subjects from Dataset A: (a,b,c), Dataset B: (d, e, f), Dataset C (g, h, i). The length of a line segment is proportional to the corresponding volume fractions; the colors indicate the orientation of fibers: medio-lateral (red), anterior-posterior (green), and superior-inferior (blue)}.

To demonstrate the performance of JARD and MLE on brain data, one subject was randomly selected from each of the three aforementioned datasets (A, B, and C). Fig 5 shows approximately corresponding coronal-views of regions of interest where the corpus callosum (CC) crosses the corticospinal tract (CST). Specifically, it contains the JARD (left) and MLE (middle) and FSL’s ARD [8] (ARD-FSL, right) estimates in this slice. From top to bottom are shown data from respectively datasets A, B, and C. A single fiber region, i.e. the central part of CC, is indicated by the yellow ellipse and the region where CC and CST cross by the green circle. For JARD and MLE, the line segments visualize the orientations of the largest eigenvectors of the underlying tensors and the length of each line segment is scaled by the volume fraction. For FSL-ARD the line segments visualize stick orientations, scaled with the estimated volume fractions. Therefore, we applied the command “bedpostX” in FSL with parameter values: number of fibers = 2, weight = 1, Burn in = 1000, and switching to multi-shell model and rician noise (leaving model noise floor off) for a comparable outcome.

Clearly, JARD forces the volume fraction of one tensor compartment nearly to zero in the single fiber region of the CC. Evidently, MLE yields an erratic outcome regarding both volume fraction and fiber orientation in the same region. Furthermore, notice that FSL-ARD often returns two fiber orientations in this region. In crossings like the region where CC crosses with CST, JARD, MLE and FSL-ARD yield a similar outcome regarding fiber orientations. These trends can be observed for each of the three datasets.

The performance of FSL-ARD is influenced by the so-called ARD weight: a higher *weight* reduces the number of secondary fibres per voxel.

**Fig 6:**
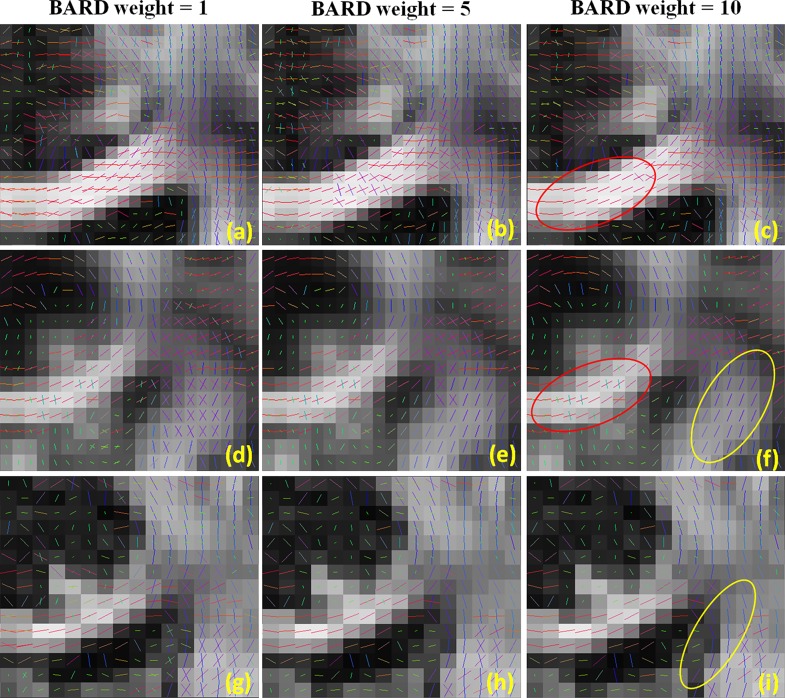
Results of FSL’s ball-and-stick parameter estimation for three different FSL-ARD weights. The ROIs and datasets are the same as in Fig 5.

Fig 6 shows the influence of the FSL-ARD weight on FSL’s performance. In this experiment, we examined the same regions as shown in Fig 5 and used three different FSL-ARD weights.

It can be noticed that a larger FSL-ARD weight reduces the number of modeled fibers, which is preferred in single fiber regions such as the central part of the corpus callosum. However, a larger weight simultaneously decreases the number of modeled fibers in crossing regions. Furthermore, the proper FSL-ARD weight varies for the different datasets: 10 for dataset A, 1 for dataset B and C. For instance, Fig 6(C) shows that weight = 10 yields good model selection in the single fiber region of Dataset A (see the red circle). However, at this same weight spurious second fibers are not effectively restrained in Dataset B (see the red circle in Fig 6(F)). On the other hand, at this weight no secondary fibers are detected in crossing regions (indicated by yellow circles in (f) and (i)). Observe that our framework (as reported in Fig 5) yields a better performance than FSL-ARD, even at the optimal weight settings, and does not require parameter tuning.

### JARD versus MLE: Fractional Anisotropy along the CC

**Fig 7:**
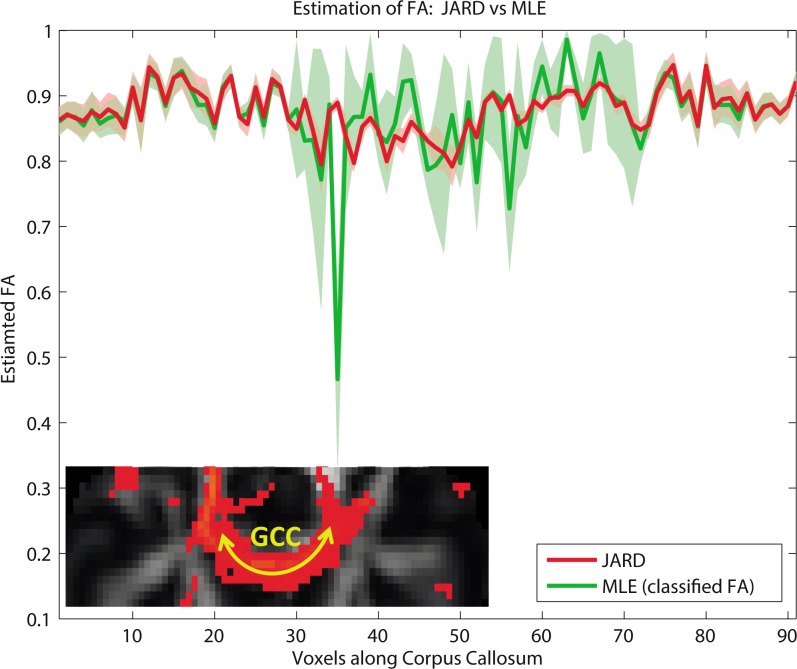
Estimated FA by JARD (red) and MLE (green) as a function of position along the genu of the corpus callosum (GCC trajectory is indicated by the yellow arrow on the inset). The estimated uncertainty (indicated by the colored background) was calculated by +/- the square root of the Cramer-Rao lower bound. The trajectory along the GCC consists of a single fiber region in the middle (30–70) and enclosed by crossing fibers region (with CST) on both ends. The figure displays the FA of the estimated tensor compartments assigned to the GCC by front evolution for both JARD and MLE.

Fig 7 shows the estimated FA along the Genu of the corpus callosum (GCC) in one subject from dataset B. The GCC is indicated by the yellow trajectory superimposed on the red-colored structure of the inset. Notice that the center of the GCC is a single fiber bundle but there is a crossing with the CST at its lateral sides. The FA along the tract was estimated by the proposed JARD framework (red) and MLE (green). We had to select the tensor compartment that corresponds to the GCC since the labels assigned by MLE and JARD are random. To solve this, the FA belonging to CC was selected based on “front evolution” [37]. In front evolution, the estimated tensor of one compartment is randomly chosen as the reference tensor. Then, the tensor of a neighborhood voxel with the smallest Frobenius norm to the reference tensor receives the same label. After processing all neighbors of the current front as such, these neighbors become the new reference tensors for the next iteration. The green (MLE) and red (JARD) areas indicate the uncertainty in the estimated value as quantified by the square root of the CRLB.

Confirming the above findings, estimation by JARD yields a rather constant FA with small variance. In contrast, MLE yields FA values with a larger variance, particularly in the central part of GCC. We attribute this to overfitting.

### JARD versus MLE: Dual-tensor FA and volume-fraction maps

FA as well as volume-fraction maps have been used to detect white matter changes [38] [37]. Here, we will display FA and volume fraction maps generated by JARD and MLE and point out the differences.

**Fig 8:**
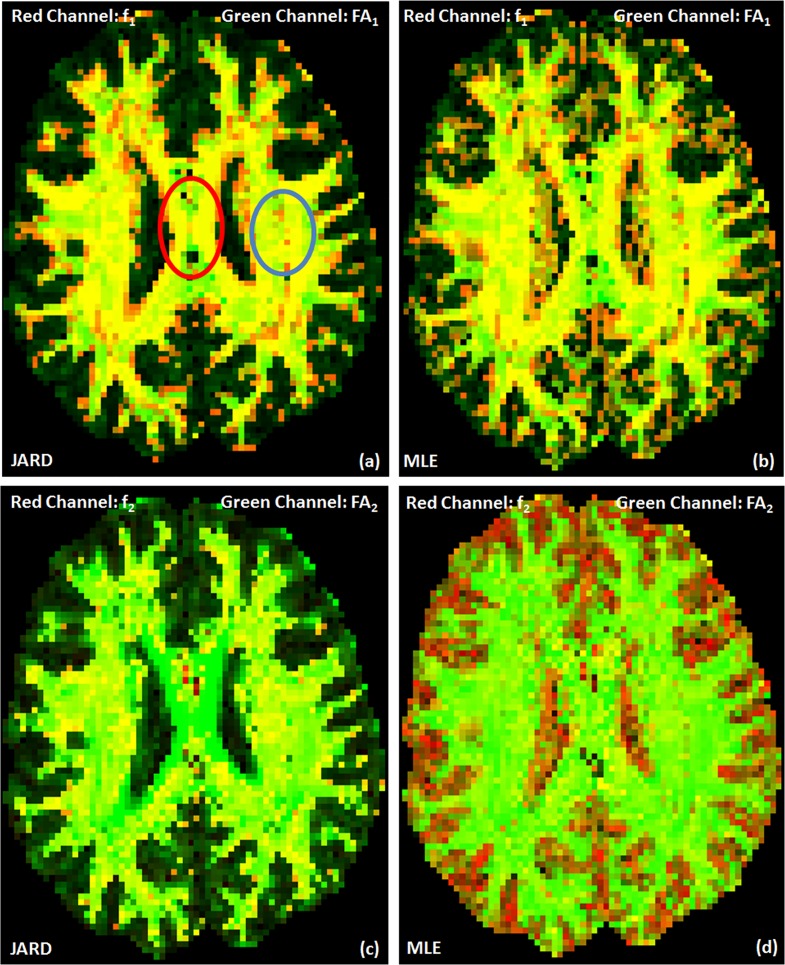
Color-coded output displaying the FA (green channel) and the corresponding volume fraction (red channel) for ARD and MLE. The tensor compartments were classified into first and second by front evolution. FA of first tensor and its corresponding volume fraction by JARD (a) and by MLE (b); FA of the second tensor and its corresponding volume fraction by JARD (c) and by MLE (d). A percentile stretch was performed on the data for more contrast. This stretching put the 5% lowest signal values to zero and the 5% highest values to 1; the remaining values were linearly mapped in between. The red circle indicates a single fiber region, the blue circle a region with crossing fibers.

Specifically, Fig 8 shows color-coded RGB maps encoding FA in the red channel and the corresponding volume fraction in the green channel. Left images show the outcome by JARD, right images by MLE; top images reflect the first tensor, bottom images denote the second tensor. The ordering of the tensors was performed by front evolution [37].

Regarding the JARD outcomes, one can observe that in Fig 8(A) and Fig 8(C) grey matter is dark, representing both small *f*_1_, *FA*_1_ and small *f*_2_, *FA*_2_. Furthermore, in Fig 8(A) single fiber regions, particularly the central part of CC, are yellowish due to simultaneously large *f*_1_ and FA_1_; in Fig 8(C) the regions are greenish because of a small *f*_2_. In Fig 8(A) crossing fiber regions are yellowish again reflecting large *f*_1_ and FA_1_; in Fig 8C) these regions are slightly more greenish, because of the smaller *f*_2_.

In contrast, MLE does not specifically force the volume fraction of one tensor to zero in single fiber and gray matter regions. Therefore, the corpus callosum in Fig 8 (D) contains more yellow spots than Fig 8(C) that reflect large *f*_2_,*FA*_2_. At the same time gray matter regions in Fig 8(B) and 8(D) contain reddish spots due to the small FA for substantial volume fractions *f*_1_, *f*_2_.

In general, Fig 8 confirms that MLE estimation is not able to automatically cope with single fiber regions whereas JARD suppresses the volume fraction of one tensor in such areas. In a region in the corpus callosum (indicated by the red circle) we measured *FA*_1_ = 0.94 ± 0.05, *f*_1_ = 0.96 ± 0.03 and *FA*_2_ = 0.93 ± 0.06, *f*_2_ = 0.04 ± 0.01 (mean ± standard deviation) using JARD. Similarly, we measured *FA*_1_ = 0.95 ± 0.04, *f*_1_ = 0.65 ± 0.12 and *FA*_2_ = 0.94 ± 0.04, *f*_2_ = 0.47 ± 0.14 after MLE. In a region with crossing fibers (indicated with the blue circle) we measured *FA*_1_ = 0.87 ± 0.02, *f*_1_ = 0.66 ± 0.01 and *FA*_2_ = 0.94 ± 0.04, *f*_2_ = 0.37 ± 0.03 using JARD. Similarly, we measured *FA*_1_ = 0.88 ± 0.02, *f*_1_ = 0.66 ± 0.02 and *FA*_2_ = 0.94 ± 0.05, *f*_2_ = 0.36 ± 0.03 using MLE.

### TBSS based on dual tensor FA and volume-fraction maps

For a statistical analysis of the FA and volume fractions with age, we included the healthy controls from dataset C [36]. The subjects aged between 45 and 50 (12 subjects, mean-age: 46.2, standard deviation 1.49) and those aged between 65 and 75 (12 subjects, mean-age: 68.2, standard deviation 3.72) were selected from the full control group. All data was registered to the MNI152 standard space using FNIRT [39]. We used the version of FNIRT that is implemented in FSL (version 5.0.7). Subsequently, differences between the two age groups were analyzed by means of the classical TBSS technique, i.e. single tensor analysis, [38] as well as the extended TBSS method for the dual tensor models [39]. Compared to the classical TBSS method, the extended TBSS technique employs ‘front evolution’ to avoid swapping of the two anisotropic components between the different images. Extended TBSS was used to analyze differences in the dual tensor volume fractions (comparable to [37]) as well as differences in the dual tensor FA maps between the two age groups. Importantly, our approach facilitates such a separate analysis of tensor volume and shape. Notice that the volume fraction used in [37] is a different variable, representing the stick strength in a ball-and-stick model.

**Fig 9:**
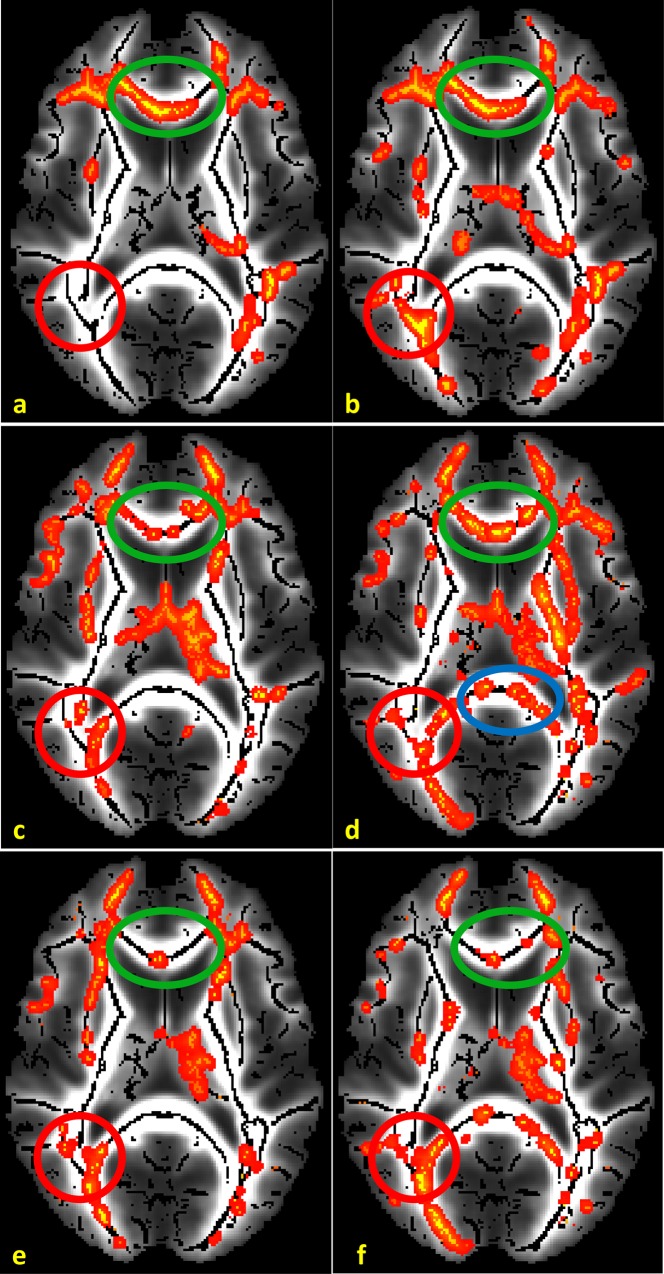
Results of TBSS on the two age groups of dataset C: classical, single tensor TBSS analysis (a); extended TBSS analysis of the volume fractions derived from FSL’s ball-and-stick model (b); extended TBSS analysis of the fiber-specific FA and volume fraction maps given by JARD (c, d) and MLE (e, f). Red-Yellow (region/tract) indicates where the FA or the volume fraction of the younger subgroup is significant larger than that of the older subgroup.

Fig 9 shows an axial slice containing multiple areas with crossing fibers and regions with just a single fiber. Fig 9(A) shows the classical, single tensor TBSS analysis and Fig 9(B) shows the extended TBSS analysis of the volume fraction estimated from FSL’s ball-and-stick model. Fig 9C and 9E contain the extended TBSS analysis of the dual tensor FA maps, and Fig 9D and 9F) the extended TBSS analysis of the dual tensor volume fraction maps. The dual tensor estimations in Fig 9C and 9D were obtained by JARD, those in Fig 9E and 9F by means of MLE. The red-yellow colored regions in Fig 9A–9F identify regions where the differences are significant.

It can be observed that the single tensor approach does not detect significant differences in the crossing fiber regions indicated by the red ellipse (Fig 9(A)). By comparison, the analysis of FSL’s ball-and-stick method yields remarkably more regions with significant differences, particularly in fiber crossings such as in the red ellipse (Fig 9(B)). The JARD method (Fig 9C and 9D)) finds slightly more significant differences compared to both the classical approach and FSL’s ball-and-stick method in single tensor regions, see the right part of the green circle (Fig 9C and 9D)) and also in the a blue circle (Fig 9(D)). This signifies that the superfluous parameters are effectively eliminated by JARD in single fiber regions. In single fiber regions the extended TBSS analysis based on MLE (Fig 9E and 9F)) yields smaller regions with significant differences compared to the classical approach (Fig 9(A)). We attribute this to the large variability in such regions that we already observed with MLE e.g. in Fig 2. Furthermore, we found the expected similarities regarding detected differences in crossing regions (e.g. the red circles) between the MLE and JARD. This indicates that Jeffreys prior does not affect the MLE outcome in such regions. All significant differences are negative, i.e. reduced FA and volume fraction with increasing age. This outcome confirms the finding that significant age-related white matter atrophy was found in the corpus callosum [40].

## Discussion

We developed a new framework for estimating the parameters of a constrained dual tensor model in diffusion-weighted MRI. It automatically determines to what extend the diffusion in a voxel should be modeled by one or two rank-2 tensors. In essence, the complexity of the model is implicitly inferred with JARD in a Bayesian probabilistic manner. An initial guess of the diffusion model is obtained by fitting a dual tensor model to the data with MLE. Subsequently, a new automated relevance determination method assesses whether two tensors are ‘mandatory’ to model the data. If this is not the case, the volume fraction of the superfluous tensor automatically reduces to (nearly) zero.

The proposed framework extends previous work by Behrens et al and Jbabdi et al [38] [37]. A crucial difference is that we employ a rank-2 tensor model, whereas the previous works concerned ball-and-stick models. As such, we aim to recover the full diffusion shape. In FSL 5.0.9, released after the initial submission of this manuscript, bedpostx was extended to include axially symmetric tensors (see http://fsl.fmrib.ox.ac.uk/fsl/fslwiki/WhatsNew#anchor1). This might reduce the number of voxels where bedpostx finds multiple fibers in the corpus callosum (as in Fig 5). However, our model still differs from this new bedpostx in two important ways: (1) bedpostx forces both tensors to have the same perpendicular diffusivity and hence FA, and (2) our prior is data-acquisition adaptive. Furthermore, an important novelty of our work is that Jeffreys non-informative prior is exploited in JARD, yielding an alternative for previously used informative priors. It facilitates accurate and precise estimation of the volume fractions as well as the diffusion properties with a DTM in single fiber and crossing fiber regions. Jeffreys prior is based on the Fisher’s information matrix which accounts for properties of the data acquisition, such as diffusion weighting *b*-value, the gradient directions and the effective SNR. Therefore we call this method data-acquisition adaptive.

We demonstrated that both in a central part of the corpus callosum (single fiber) as well as in a region where the corpus callosum crosses with the corticospinal tract, the configuration inferred by our method corresponds to the expected neuro-anatomy [41] [42]. The proposed framework has been compared with direct MLE of the same dual tensor model. Several differences between the proposed framework and MLE were observed. In regions that were considered to contain just a single fiber, MLE typically inferred a large volume fraction for both tensor components (see Fig 5). Here, the proposed framework yielded a single tensor representation by diminishing the volume fraction of the second tensor component. Furthermore, the FA estimated by MLE showed much more variation than the FA estimated by JARD in such a region (Fig 7). In regions that were considered to contain crossing fibers, the results of MLE and JARD were similar.

There are a few limitations of our method. Firstly, we assume a mono-exponential decay along the eigenvectors of the three compartments up to *b* = 3000 s/mm^2^. Measuring at higher *b*-values will certainly introduce sensitivity to different compartments such as the myelin sheet [33] with the associated restricted and hindered diffusion [5]. In the latter case, the Gaussian diffusion assumption is no longer valid. However, investigating such non-Gaussian diffusion is beyond the scope of this work. As such, we follow [3] and [43]. Secondly, recent studies reported the presence of a three-way crossing of fiber bundles [14] [44]. In our framework a dual-tensor model is employed to characterize voxels encompassing crossing fibers. The reason for limiting the number of anisotropic components to two is the limited SNR in our HARDI data. Notice that whereas [14] and [44] only recover the fiber orientation, we aim to reconstruct the full diffusion shape, which requires a higher SNR. In [3] we showed that estimating a dual rank-2 tensor model already requires HARDI at two *b*-values, data of sufficient SNR, and some model restrictions. The latter is needed to ensure stability as the number of model parameters may approach or even surpass the number of degrees of freedom present in the data. Therefore, fitting a triple rank-2 tensor model to voxels with a three-way-crossing will be even more challenging [13]. Developing methods for estimation of the diffusion properties in three-way-crossing fiber bundles will remain an important challenge for future research.

Diffusion imaging may reveal several aspects to white matter integrity: (I) locations of alterations; (II) which fiber tract is affected; (III) the exact change in diffusion. Previously, many solutions were already proposed for the first two aspects. Our work focused on all three aspects. Particularly, our framework may aid a more accurate characterization of the diffusion shape.
